# Effects of intra-abdominal sepsis on atherosclerosis in mice

**DOI:** 10.1186/s13054-014-0469-1

**Published:** 2014-09-03

**Authors:** Ata Murat Kaynar, Sachin Yende, Lin Zhu, Daniel R Frederick, Robin Chambers, Christine L Burton, Melinda Carter, Donna Beer Stolz, Brittani Agostini, Alyssa D Gregory, Shanmugam Nagarajan, Steven D Shapiro, Derek C Angus

**Affiliations:** The Clinical Research, Investigation, and Systems Modeling of Acute Illness (CRISMA) Center, University of Pittsburgh, Scaife Hall 612, 3550 Terrace Street, Pittsburgh, PA 15261 USA; Department of Critical Care Medicine, University of Pittsburgh, Pittsburgh, PA USA; Current address: Shengjing Hospital, China Medical University, Shenyang, Liaoning Province, China; Current address: Department of Biology, Tulane University, New Orleans, LA USA; Current address: Pittsburgh Zoo and PPG Aquarium, Pittsburgh, PA USA; Department of Medicine, University of Pittsburgh, Pittsburgh, PA USA; Department of Cell Biology, University of PittsburghAquarium, Pittsburgh, PA USA

## Abstract

**Introduction:**

Sepsis and other infections are associated with late cardiovascular events. Although persistent inflammation is implicated, a causal relationship has not been established. We tested whether sepsis causes vascular inflammation and accelerates atherosclerosis.

**Methods:**

We performed prospective, randomized animal studies at a university research laboratory involving adult male ApoE-deficient (ApoE^−/−^) and young C57B/L6 wild-type (WT) mice. In the primary study conducted to determine whether sepsis accelerates atherosclerosis, we fed ApoE^−/−^ mice (*N* = 46) an atherogenic diet for 4 months and then performed cecal ligation and puncture (CLP), followed by antibiotic therapy and fluid resuscitation or a sham operation. We followed mice for up to an additional 5 months and assessed atheroma in the descending aorta and root of the aorta. We also exposed 32 young WT mice to CLP or sham operation and followed them for 5 days to determine the effects of sepsis on vascular inflammation.

**Results:**

ApoE^−/−^ mice that underwent CLP had reduced activity during the first 14 days (38% reduction compared to sham; *P* < 0.001) and sustained weight loss compared to the sham-operated mice (−6% versus +9% change in weight after CLP or sham surgery to 5 months; *P* < 0.001). Despite their weight loss, CLP mice had increased atheroma (46% by 3 months and 41% increase in aortic surface area by 5 months; *P* = 0.03 and *P* = 0.004, respectively) with increased macrophage infiltration into atheroma as assessed by immunofluorescence microscopy (0.52 relative fluorescence units (rfu) versus 0.97 rfu; *P* = 0.04). At 5 months, peritoneal cultures were negative; however, CLP mice had elevated serum levels of interleukin 6 (IL-6) and IL-10 (each at *P* < 0.05). WT mice that underwent CLP had increased expression of intercellular adhesion molecule 1 in the aortic lumen versus sham at 24 hours (*P* = 0.01) that persisted at 120 hours (*P* = 0.006). Inflammatory and adhesion genes (tumor necrosis factor α, chemokine (C-C motif) ligand 2 and vascular cell adhesion molecule 1) and the adhesion assay, a functional measure of endothelial activation, were elevated at 72 hours and 120 hours in mice that underwent CLP versus sham-operations (all at *P* <0.05).

**Conclusions:**

Using a combination of existing murine models for atherosclerosis and sepsis, we found that CLP, a model of intra-abdominal sepsis, accelerates atheroma development. Accelerated atheroma burden was associated with prolonged systemic, endothelial and intimal inflammation and was not explained by ongoing infection. These findings support observations in humans and demonstrate the feasibility of a long-term follow-up murine model of sepsis.

**Electronic supplementary material:**

The online version of this article (doi:10.1186/s13054-014-0469-1) contains supplementary material, which is available to authorized users.

## Introduction

Several studies have suggested a link between acute cardiovascular events (such as cardiac event–related death, acute myocardial infarction and stroke) and prior infection [1-4]. However, a lack of detailed measures of cardiovascular disease burden prior to the occurrence of infection, particularly subclinical disease, has the potential to confound the interpretation of these studies [1-3]. Furthermore, the mechanisms underlying this association are unclear. Ongoing infection in vessel walls was first thought to be the cause of accelerated atherosclerosis, but this link has never been established [4]. We previously demonstrated that human sepsis survivors often have persistent inflammation after the infection has resolved, which is associated with an increased risk of subsequent cardiovascular death [5]. We therefore hypothesized that sepsis may lead to persistent vascular inflammation, which in turn could accelerate the growth or destabilization of atheromatous plaques. In other words, we hypothesized that the dysregulated immune response characteristic of sepsis may have a persistent “tail” that accelerates the progression of underlying cardiovascular disease [6].

We wished to test this hypothesis in experimental animal models by using a combination of existing murine models of atherosclerosis and sepsis. Our work was focused on both short- and long-term effects of sepsis, in contrast to prior studies that focused only on short-term inflammatory changes following viral infections [7].

## Material and methods

### Study overview

**Figure 1:**
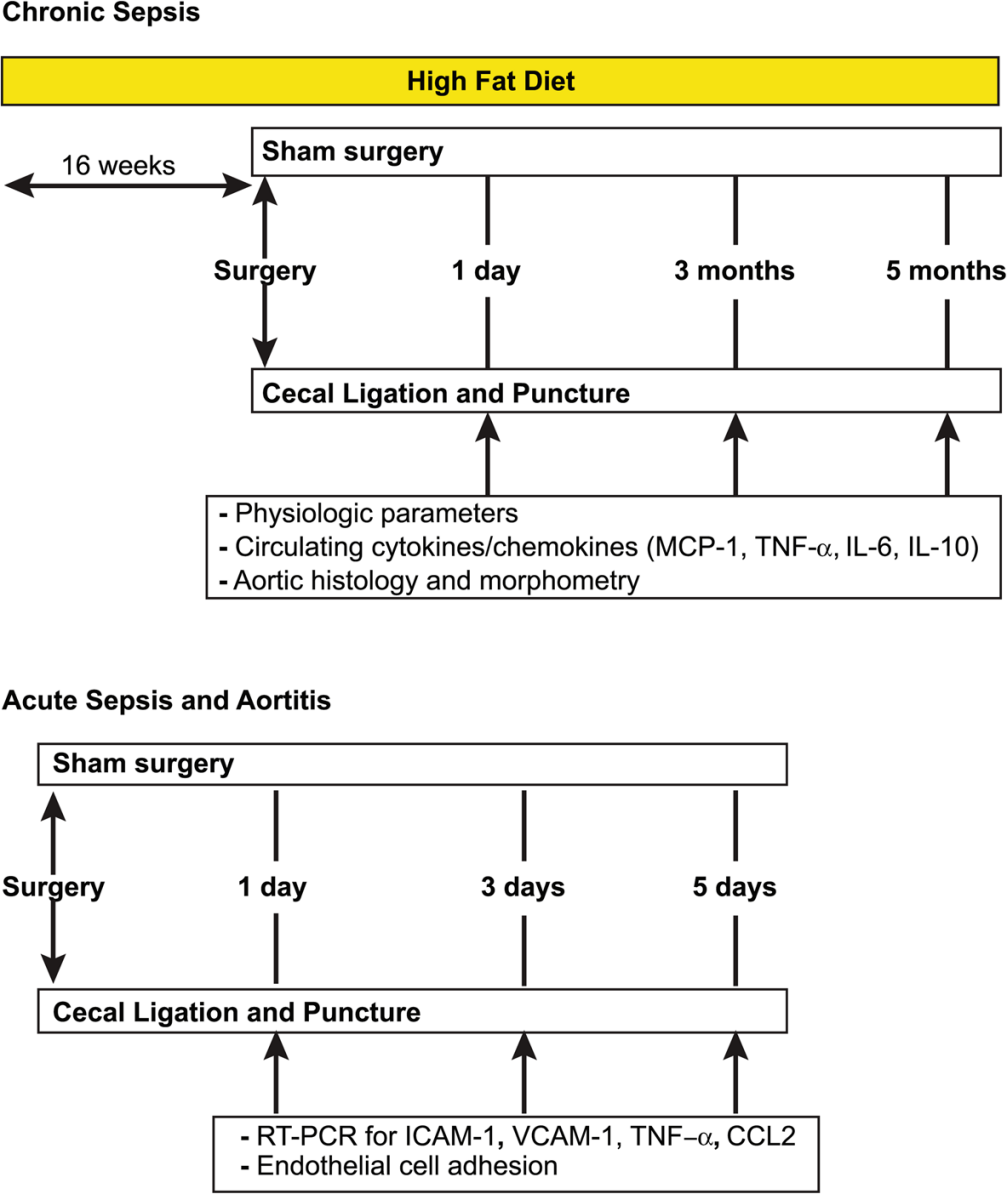
**Time scale of the experimental protocol.** In the primary experimental protocol, a sepsis survival program was designed in apolipoprotein E–deficient mice fed a high-fat diet prior to the surgical intervention. In the secondary acute model, the wild-type mice were fed a regular diet until the time of surgery. CCL2, Chemokine (C-C motif) ligand 2; ICAM-1, Intercellular adhesion molecule 1; IL, Interleukin; MCP-1, Monocyte chemotactic protein 1; TNF-α, Tumor necrosis factor α; VCAM-1, Vascular cell adhesion molecule 1.

In our primary experiment, we assessed whether sepsis has long-term effects on physical function, systemic inflammation and atheroma in animals with preexisting cardiovascular disease. To mimic chronic preexisting cardiovascular disease, we used the well-established model of ApoE-deficient (ApoE^−/−^) mice fed an atherogenic diet for 16 weeks [8]. In this model, atheroma burden in the aorta is considered a mimic of human coronary artery disease. To mimic sepsis, we then performed cecal ligation and puncture (CLP), a well-established model of sepsis [9]. We gave these mice intraperitoneal fluids and antibiotics to mimic clinical practice and adjusted the needle size in pilot experiments until the day 7 lethality was low. We then randomly assigned 46 atherogenic diet–fed ApoE^−/−^ mice to the CLP or sham operation group and followed them for up to 5 months. We monitored weight and physical activity over time and killed the animals at select time points to assess circulating inflammatory markers and atheroma burden via aortic morphometry, histology and immunofluorescent staining for macrophages. In a secondary experiment, we exposed 32 young wild-type (WT) mice to CLP or a sham operation with 5-day follow-up to determine the immediate effects of sepsis on aortic wall inflammation (Figure 1). All aspects of this study complied with the Guide for the Care and Use of Laboratory Animals published by the National Institutes of Health (NIH Publication 85-23 (revised 1996)) and met the approval of the Institutional Animal Care and Use Committee of the University of Pittsburgh (IACUC 1110706B-2).

### Primary experimental protocol: atherosclerosis in ApoE^−/−^ mice followed by CLP or sham surgery

We first conducted pilot studies to establish the combined model. To generate a model of atherosclerosis, we obtained 6- to 8-week-old male ApoE^−/−^ mice on a C57BL/6 background from The Jackson Laboratory (Bar Harbor, ME, USA), housed them in pathogen-free rooms and fed them a standard atherogenic Western diet (Teklad TD.88137; Harlan Laboratories, Indianapolis, IN, USA) for 16 weeks [10]. We killed 8 animals after 16 weeks and confirmed that there were macroscopically visible atherosclerotic lesions in the aortic root and descending aorta. These plaques were yellowish-white in appearance, projected into the lumen of the aorta and were more abundant in the arch of the aorta as well as in the iliac arteries, sites of turbulent flow. To generate a model of sepsis that mimicked the clinical scenario of severe infection from which animals had a strong likelihood of surviving with supportive care, we pilot-tested alternative needle sizes and supportive care strategies in the ApoE^−/−^ atherogenic diet–fed mice. In the final pilot in 16 mice, we used a 25-gauge needle with a single perforation, followed by fluid resuscitation and antibiotics at 12, 36 and 60 hours after the surgery. This led to notable limitation of physical activity (see Additional file 1). Only one animal died by day 7.

Following the pilot phase, we randomized forty-six 22- to 24-week-old, atherogenic diet–fed ApoE^−/−^ mice to the sham operation or CLP group (Figure 1). For sham or CLP surgery, mice received intraperitoneal anesthesia with ketamine (85 mg/kg) and xylazine (13 mg/kg). We performed a left-sided abdominal paramedian vertical incision (about 2 cm). In the sham-operated group, we mobilized the cecum and then closed the abdomen. In the CLP group, we mobilized the cecum, ligated approximately 40% of its length from the base without compromising the blood flow, and punctured with a 25-gauge needle. A small amount of fecal contents were expressed, then the cecum was placed back into the peritoneum and the abdomen was closed in layers. A single operator performed all of the surgical procedures to eliminate interoperator variability. We then injected 1 ml of sterile warm saline intraperitoneally to both groups. Both groups of mice received intraperitoneal imipenem-cilastatin (Merck, Whitehouse Station, NJ, USA) (25 mg/kg) and saline solution at 12, 36 and 60 hours after the surgery. All mice received diet, water and DietGel (ClearH_2_O, Portland, ME, USA) *ad libitum* and were monitored at least twice daily.

Mice were killed per protocol on day 1 and at 3 and 5 months to obtain aortic tissue samples and blood for circulating inflammatory markers (Figure 1). We decided on these time points based on previously published literature suggesting a progressive increase in atherosclerosis in ApoE^−/−^ mice over the course of 6 months [11]. We killed 16 animals (8 per group) on day 1, 13 (6 in the CLP group and 7 sham-operated) at 3 months and the remainder (9 in the CLP group and 8 sham-operated) at 5 months. Of note, animals would be killed early if they became moribund. However, all animals reached the per-protocol time points.

#### Clinical parameters

Following sham or CLP surgery in ApoE^−/−^ mice, we measured body weight and activity at days 0, 1, 2, 7 and 14 using a score previously validated by Zantl *et al*. [12-14]. The score is a composite index of overall well-being of septic mice. The index is composed of fur quality, weight change, baseline and evoked activity as a surrogate for disease severity, used both during the pilot and actual experiments. It was not possible to blind the assessments, as the CLP mice had an obvious decrease in physical activity.

#### Aortic histology and morphometry to study progression of atheroma

We conducted histologic and morphometric assessments of the descending aorta and aortic root to assess atherosclerotic burden in ApoE^−/−^ mice. We flushed the arterial system with cold phosphate-buffered saline (PBS) via the left ventricle, excised aortae from the arch to the iliac bifurcation and placed them in cold PBS. We removed adventitial fat and opened aortae longitudinally. To determine atheroma in the descending aorta, we next placed dissected aortae in 70% ethanol and stained the sections with oil red O (90 minutes) (Sigma-Aldrich, St Louis, MO, USA) and mounted them *en face* in glycerol-gelatin mounting medium on a polymer board (Sigma-Aldrich). We scanned the aortae with an Olympus macroscope (Olympus, Center Valley, PA, USA), analyzed them using MetaMorph (Molecular Devices, Sunnyvale, CA, USA) and calculated atheroma burden as the percentage of total aortic area. To determine aortic root atheroma, we obtained 7-μm-thick sections and stained them with hematoxylin and eosin for structural morphology, oil red O for atheroma burden and Sirius Red for fibrillar collagen. To measure both descending and root aorta plaque sizes, we used MetaMorph computer-assisted image analysis software to determine stained areas with consistent thresholds across all the image fields. Because the descending aorta was intact, we expressed atheroma as the percentage of total surface area. For the aortic root, which was analyzed cross-sectionally, we present data as both absolute values and adjusted for mouse weight [10].

We determined the topographic relationship between atheroma progression and macrophages in ApoE^−/−^ mice using immunofluorescent staining with Mac-3 antibody. We focused on the macrophages because macrophages play an important role in sepsis and atherosclerosis [15]. We fixed the aortic root and ascending aorta in 2% paraformaldehyde in PBS for 2 hours, placed them in 30% sucrose for 24 hours, embedded them in optimal cutting temperature (OCT) compound, stored them at −80°C and sectioned them (7 μm) with a cryostat. Prior to blocking nonspecific binding sites, we permeabilized samples in 0.1% Triton X-100 in PBS for 10 minutes and then washed them in PBS. Tissues were quenched with H_2_O_2_ for 10 minutes at room temperature and then blocked for nonspecific binding with 2% bovine serum albumin (BSA) for 1 hour. We detected macrophages using rat monoclonal anti-mouse Mac-3 antibody (1:100, clone M3/84; BD Biosciences, San Diego, CA, USA). Secondary antibody mixtures of horse anti-mouse immunoglobulin G (IgG) coupled to Texas Red and goat anti-rabbit IgG conjugated with fluorescein isothiocyanate (FITC) were used. To correlate the localization of different antigens and connective tissue components, we took images of the same microscopic fields with each filter set (FITC, Texas Red and 4′,6-diamidino-2-phenylindole dihydrochloride (DAPI)) and merged them using MetaMorph software.

#### Circulating inflammatory cytokines

To determine whether inflammation persists during recovery, we measured serum tumor necrosis factor α (TNF-α), interleukin 6 (IL-6), IL-10 and monocyte chemotactic protein 1 (MCP-1) on day 1 and at 3 and 5 months after sham or CLP surgery in ApoE^−/−^ mice. Cytokines were measured with multiplex bead-based Luminex assays (R&D Systems, Minneapolis, MN, USA) and analyzed with a Bio-Plex™ 200 instrument and Bio-Plex™ Manager software (Bio-Rad Laboratories, Hercules, CA, USA). The lower limits of detection for the inflammatory cytokines were as follows: TNF-α, 2.52 pg/ml; IL-6: 3.48 pg/ml; IL-10: 2.67 pg/ml; and MCP-1, 2.21 pg/ml.

### Secondary experimental protocol: aortic wall inflammation in young WT C57BL/6 mice following CLP or sham surgery

To understand whether sepsis has acute inflammatory effects on vessel walls, we also conducted a simpler model of CLP with short follow-up in WT animals receiving a regular diet without prior induction of atheroma. Using thirty-two 6- to 8-week-old male C57BL/6 WT mice, we administered the same animal housing protocol, CLP or sham procedures and sample collection as we used in ApoE^−/−^ mice. We killed 6 animals (three in each group) on day 1, 16 (7 CLP and 9 sham-operated mice) on day 3 and the remainder (4 CLP and 6 sham-operated mice) on day 5.

To explore aortic inflammation in the early phases of sepsis, we examined protein and mRNA expression of inflammatory markers (MCP-1 and TNF-α) and adhesion molecules (intercellular adhesion molecule 1 (ICAM-1) and vascular cell adhesion molecule 1 (VCAM-1)) and endothelial activation in the aorta on days 1, 3, and 5 after sham or CLP surgery in WT mice. We focused on these markers because they play an important role in atherosclerosis [16].

To compare inflammatory marker and adhesion molecule protein expression (VCAM-1, ICAM-1, TNF-α and MCP-1), we used immunofluorescent staining. We cut the ascending aorta away from the heart at an angle to include the aortic valve, placed it into 2% paraformaldehyde in PBS for 2 hours and then put it into a 30% sucrose solution for 24 hours. Next, we embedded aortic tissues in OCT compound, maintained them at −80°C and transversely cut (7 μm thick) them to include the aortic valves. The frozen sections were then left to dry at room temperature for 30 minutes. Once dried, the slides were placed into a 2% BSA solution at room temperature for 1 hour. Following the 1-hour blocking step, the tissues were incubated with ICAM-1 (1:100, AF796; R&D Systems) and VCAM-1 (1:50, 14-1061-81; eBioscience, San Diego, CA, USA) in a 0.5% BSA solution at 4°C overnight. All of the subsequent incubations were performed at room temperature. The primary antibodies were washed thoroughly with PBS, and the secondary antibodies (anti-goat Alexa Fluor 488 and anti-rat Alexa Fluor 594; 1:500) were each incubated in a 0.5% BSA solution for 1 hour. The slides were washed with PBS and mounted in VECTASHIELD mounting medium (Vector Laboratories, Burlingame, CA, USA) containing DAPI as a nuclear counterstain and then examined with a Nikon confocal microscope (Nikon Instruments, Melville, NY, USA).

We performed RT-PCR to study gene expression for inflammatory markers and adhesion molecules (VCAM-1, ICAM-1, TNF-α and chemokine (C-C motif) ligand 2 (CCL2)) in the aortic arch. The correlation between the locations of atherosclerotic lesions and regions of disturbed flow is well documented for the aortic arch, and the murine aortic arch has flow dynamics and gene expression patterns similar to human aortic arch tissues [15,17,18]. Aortic arches were homogenized in TRIzol reagent, and total RNA was extracted according to the manufacturer’s instructions (Ambion, Austin, TX, USA). RNA yield from individual mice was 0.6 to 1.0 μg per aortic arch. Single-stranded cDNA was synthesized using the High-Capacity cDNA Reverse Transcription Kit (Applied Biosystems, Foster City, CA, USA), and RT-PCR was then performed using TaqMan Universal PCR Master Mix (Applied Biosystems). The reaction volume (25 μl) consisted of 12.5 μl of 2× TaqMan Universal PCR Master Mix, 1 μl of cDNA and 1.25 μl of 20× 6-carboxyfluorescein-labeled TaqMan Gene Expression Assay Master Mix solution. For the real-time PCRs of the genes (VCAM-1, ICAM-1, CCL2, TNF-α and β-actin), the TaqMan inventoried Assays-on-Demand Gene Expression products were purchased from Applied Biosystems. The fold difference in expression of target cDNA was determined using the comparative threshold cycle method as described previously [19]. The primers used were as follows: VCAM-1: ATGCACCAAGTACAAAGTCAGC and TTGGTCGAACTCAGGATTAGC (202 bp); ICAM-1: CTGAACATCCCATGACCTTCC and GCCCAAGGACATATTCACAGC (209 bp); CCL2: TGACCAGTGCCCATGACAAGC and CATTGTTCCCGTGCATCAAAGG (229 bp); TNF-α: TACCAGCTCCCAAAATCCTG and TCTGCTAATTCCAGCCTCGT (152 bp); β-actin: GTGATCCCTGGGCCTGGTG and GGAAACGAATACACGGTGATGG (278 bp).

To explore early endothelial activation, we used an *ex vivo* monocyte adhesion assay. We dissected the descending aorta from the arch to the iliac bifurcation and placed it into complete cell culture medium containing Dulbecco’s modified Eagle’s medium with 10% fetal bovine serum. We used RAW-Blue cells (InvivoGen, San Diego, CA, USA) as the monocyte cell line to observe adhesion, and cells between passages 6 and 15 were used. RAW-Blue cells were labeled with the acetomethoxy derivative of calcein (1 μM), and adhesion to the mouse aorta was carried out as described previously [20,21]. Photographs were taken using a fluorescence macroscope (Carl Zeiss, Thornwood, NY, USA), and the number of macrophages that adhered to the mouse aorta were counted using the MetaMorph imaging analysis system (Molecular Devices) and normalized to the total aortic area.

### Statistical analysis

Data are presented as mean ± SD. Statistical analysis was carried out using two-way analysis of variance for weight changes and activity for continuous variables, and a nonparametric Mann-Whitney *U* test was used for the other parameters measured. We defined statistical significance as *P* < 0.05.

## Results

### Long-term clinical effects of sepsis

**Figure 2:**
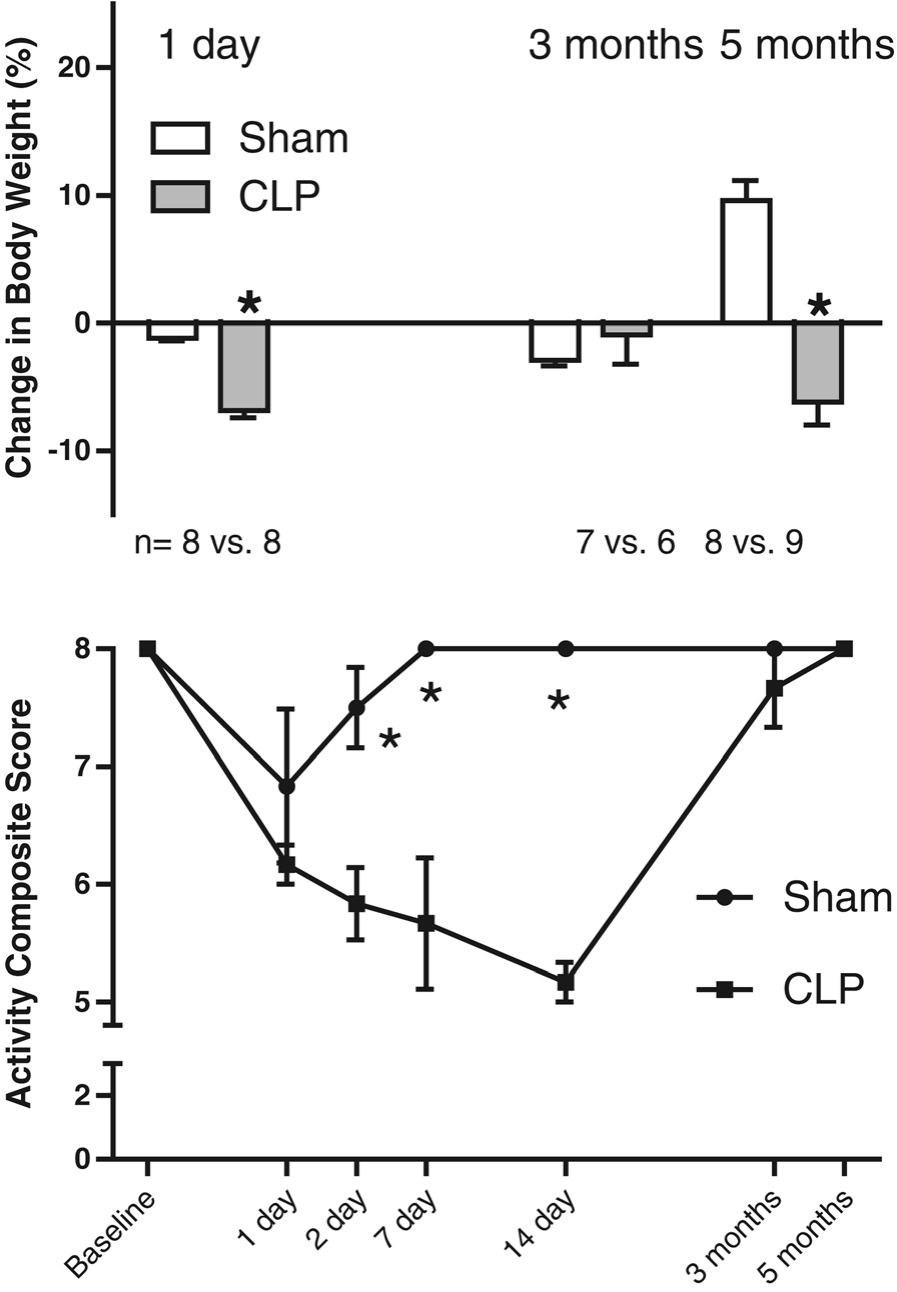
**Change in body weight and activity composite score over the course of 5 months in the primary experimental protocol, chronic sepsis survival model.** The analysis was performed in animals that underwent sham or cecal ligation and puncture (CLP) surgery (six per time point and nine per time point, respectively) and analyzed by two-way analysis of variance (*P < 0.001).

Following CLP, the ApoE^−/−^ mice had a delayed return to activity and failed to thrive compared to the sham-treated group (Figure 2). CLP mice were less active in the first 2 weeks after surgery compared to the sham-operated groups (8+/−0 vs. 5.1+/−0.4 Activity Composite Index for sham vs. CLP; *P* < 0.001), but the activity was not different between the groups by 3 months. Weight gain, however, remained different up to 5 months, when the CLP group had lost 6% of body weight compared to a 9% gain among the sham-operated mice (*P* < 0.001).

### Long-term effects of sepsis on atheroma burden

**Figure 3:**
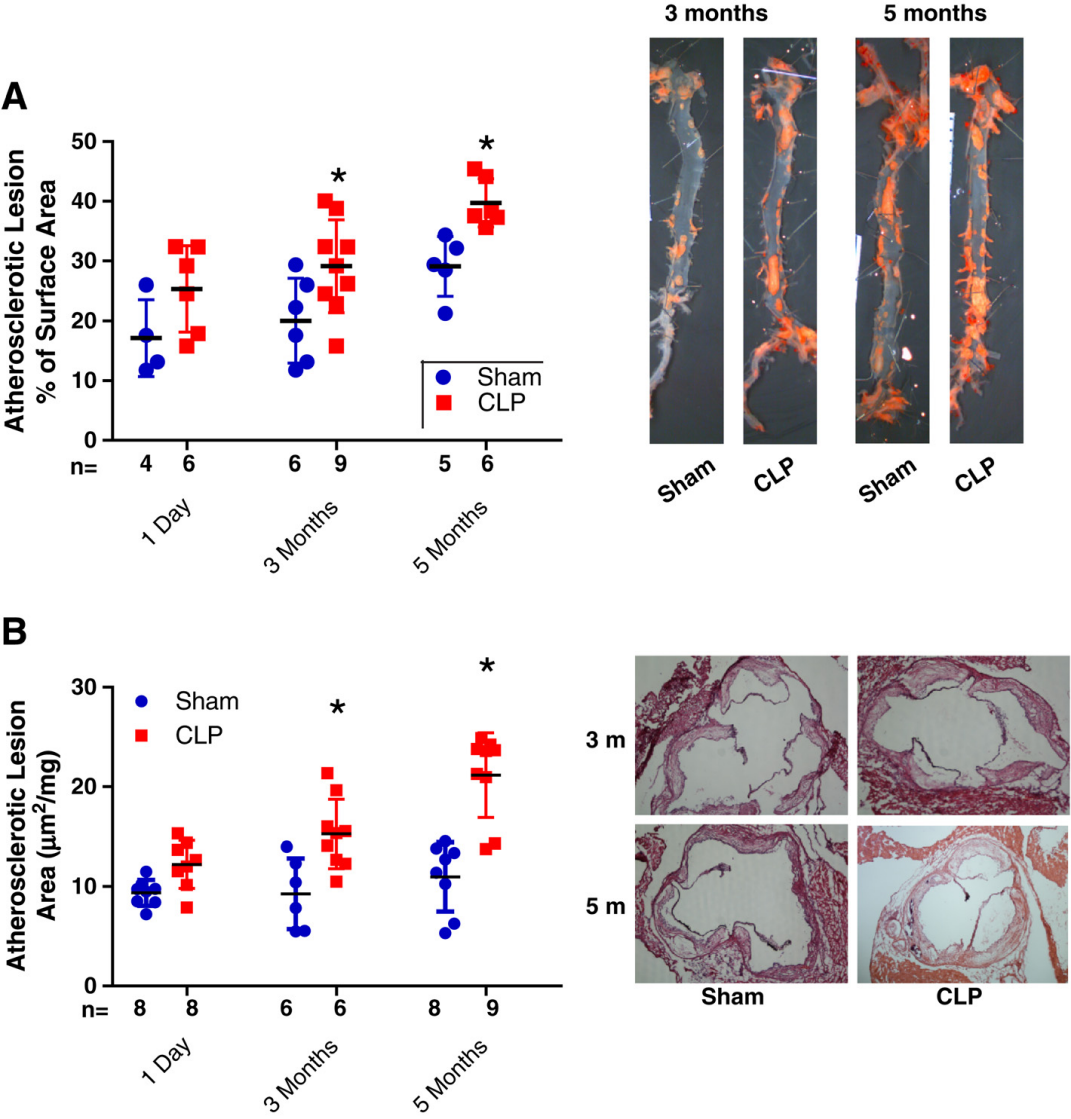
**Atheroma burden in the descending aorta and aortic root in association with sepsis and duration of chronic inflammation. (A)** Representative images of descending aorta with atheroma burden. The atheroma burden was recorded at 1 day, 3 months (3 m) and 5 months (5 m) after surgery. **(B)** Representative images of aortic root atheroma burden at similar time points. *P < 0.05 compared to the time-matched sham-treated groups. CLP, Cecal ligation and puncture.

**Figure 4:**
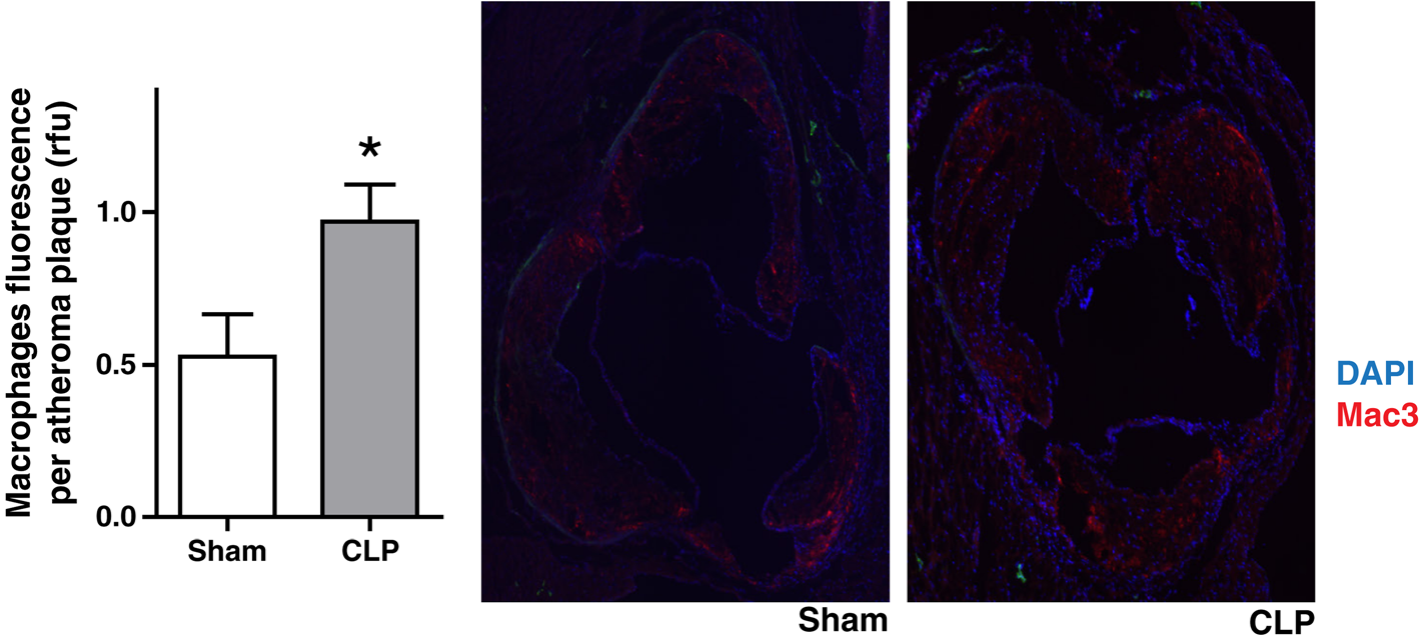
**Aortic root macrophage infiltration by 5 months suggestive of chronic inflammatory cell infiltration into the plaques.** Macrophages are shown in red and nuclear material in blue (4′,6-diamidino-2-phenylindole dihydrochloride (DAPI)). The relative fluorescence (rfu) per atheroma is presented at the 5-month time point (*P < 0.05). CLP, Cecal ligation and puncture.

The aortic atheroma burden in the descending aorta increased over the course of the experimental period in both the sham- and CLP-treated ApoE^−/−^ mice, but it was more pronounced in the CLP-treated animals at both 3 months (20% vs. 29.2% of total aortic surface area; *P* = 0.03) and 5 months (28.1% vs. 39.7% of total aortic surface area; *P* = 0.004) after surgery (Figure 3A). Similarly, CLP mice at 3 months had an increased atheroma burden at the aortic root (0.129 mm^2^ vs. 0.263 mm^2^; *P* = 0.03). By 5 months, the unadjusted atheroma burden was 0.31 mm^2^ in sham-operated mice and 0.197 mm^2^ in the CLP mice (*P* = 0.006), but, normalized for weight, it was higher in the CLP group (11.6 μm^2^/mg vs. 20.1 μm^2^/mg; *P* = 0.005) (Figure 3B). Collagen deposition was similar in both sham- and CLP-treated animals at 5 months (38.5% vs. 54.3% of atheroma plaque area for sham vs. CLP; *P* > 0.05) (see Additional file 2), but macrophage infiltration was significantly elevated at 5 months in the CLP group (0.52 vs. 0.96 relative fluorescence units; *P* = 0.038) (Figure 4).

### Long-term effects of sepsis on circulating inflammatory markers

**Figure 5:**
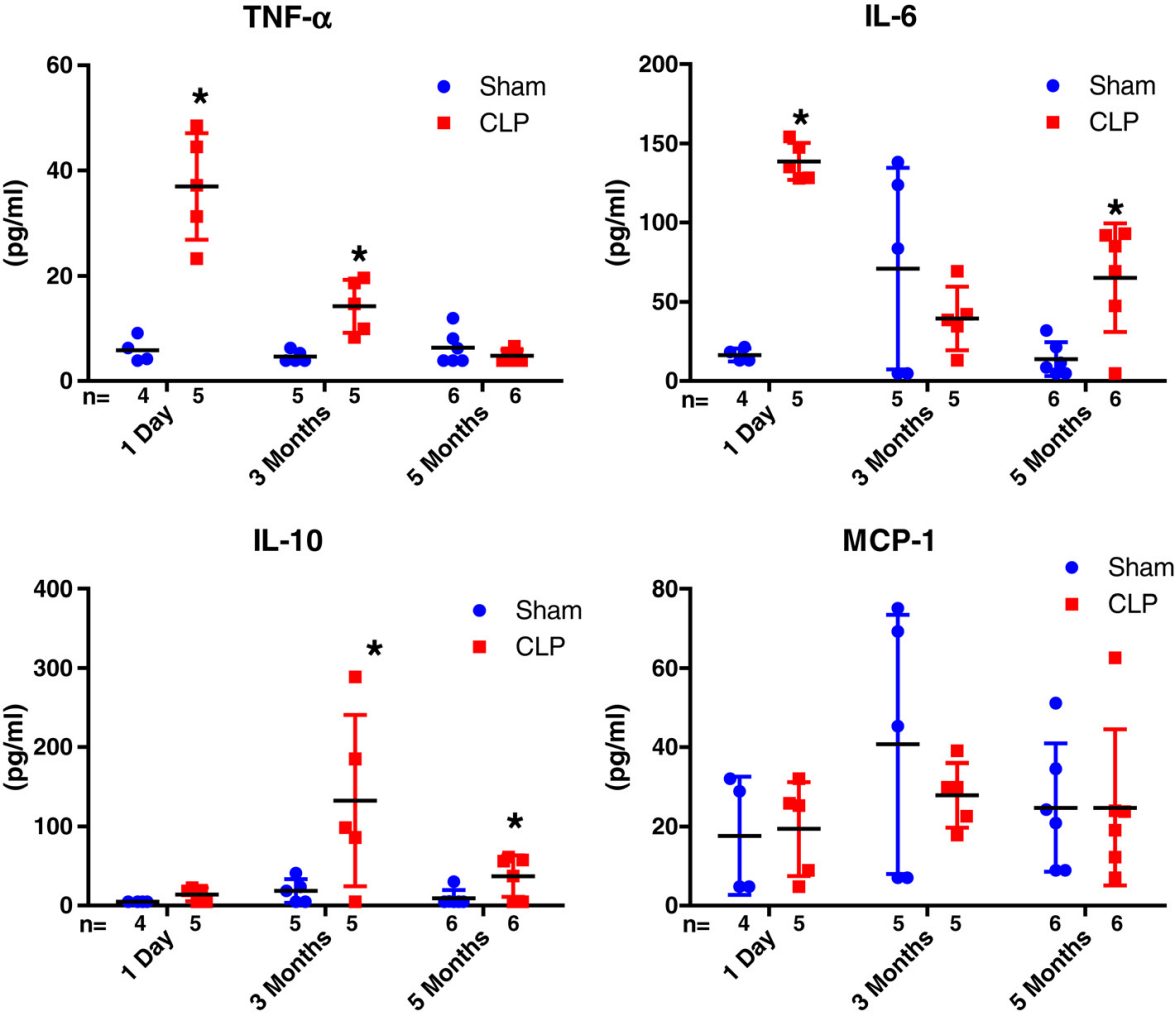
**The changes in the circulating cytokine levels at 1 day, 3 months and 5 months after surgery in apolipoprotein E–deficient mice.** The cytokines were tumor necrosis factor α (TNF-α), interleukin (IL-6) and IL-10 and monocyte chemotactic protein 1 (MCP-1). *P < 0.05 compared to the time-matched sham-operated group. CLP, Cecal ligation and puncture.

All peritoneal cultures at 3 and 5 months were sterile in both CLP- and sham-operated ApoE^−/−^ mice. As shown in Figure 5, the proinflammatory cytokines TNF-α and IL-6 were higher on day 1 in the CLP group (TNF-α: 5.8 pg/ml vs. 36.9 pg/ml, *P* = 0.002; IL-6: 16.4 pg/ml vs. 138.7 pg/ml, *P* < 0.001 (both for sham vs. CLP)), though IL-10 was not different (4.8 pg/ml vs. 13.9 pg/ml, *P* = 0.07). Several cytokines remained elevated up to 5 months (IL-6: 13.06 pg/ml vs. 65.3 pg/ml, *P* = 0.005; IL-10: 9.13 pg/ml vs. 37.11 pg/ml, *P* = 0.035 (both for sham vs. CLP)), although TNF-α and MCP-1 were no longer different.

### Early effects of sepsis on inflammatory gene expression and function in the aorta

**Figure 6:**
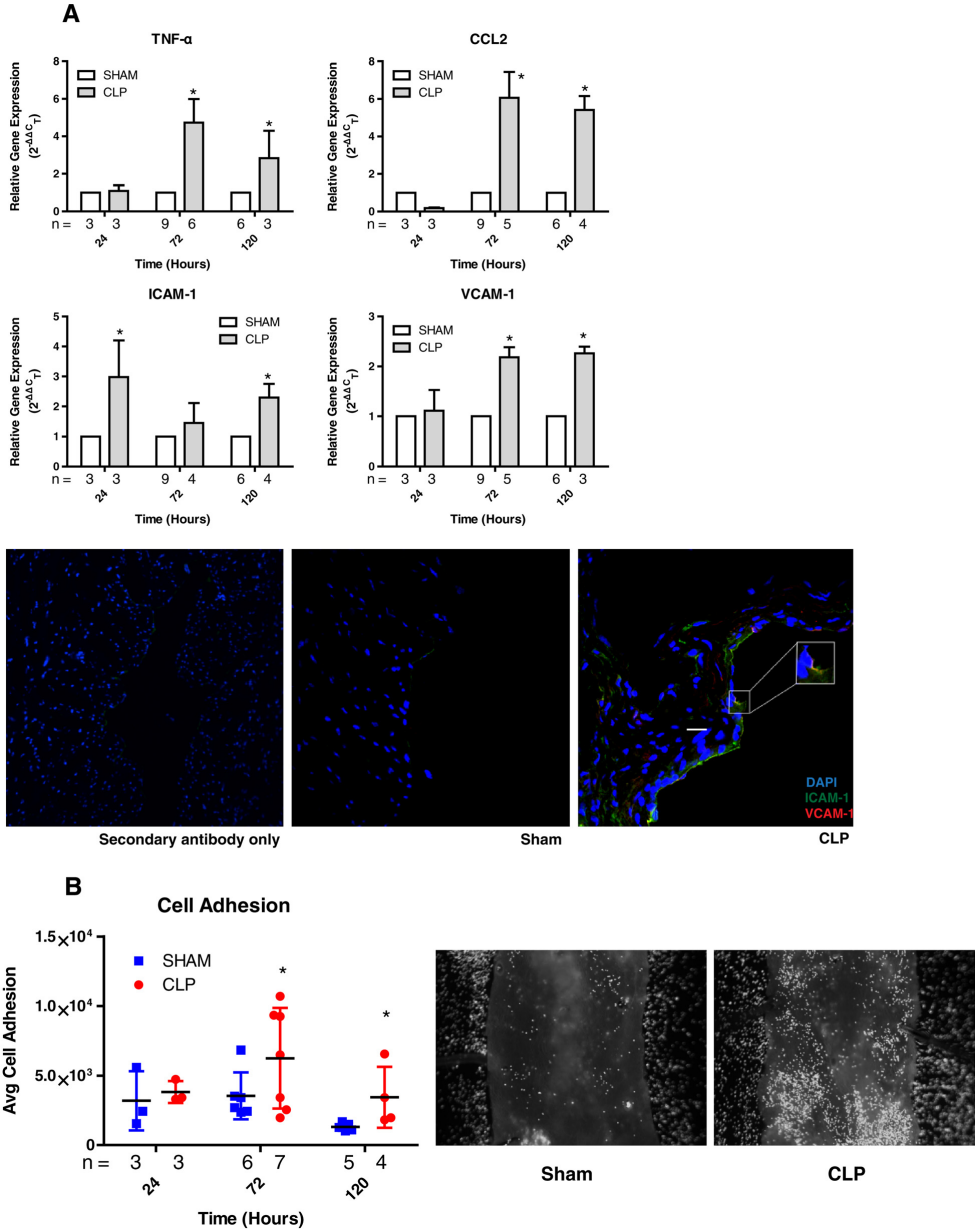
**Changes in aortic root and aortic arch mRNA expression for cytokines and adhesion molecules (tumor necrosis factor α, chemokine (C-C motif) ligand 2, intercellular adhesion molecule 1 and vascular cell adhesion molecule 1) determined using RT-PCR in wild-type mice. (A)** Relative expression at days 1, 3 and 5 days are presented. CCL2, Chemokine (C-C motif) ligand 2; CLP, Cecal ligation and puncture; TNF-α, Tumor necrosis factor α. Immunofluorescent images of the aortic root obtained to determine the expression of adhesion molecules intercellular adhesion molecule 1 (ICAM-1) and vascular cell adhesion molecule 1 (VCAM-1) (left to right: secondary antibody–only control, sham, cecal ligation and puncture (CLP)). ICAM-1 is shown in green, VCAM-1 in red and nuclear material in blue (4′,6-diamidino-2-phenylindole dihydrochloride (DAPI)). Inset shows possible colocalization of ICAM-1 with VCAM-1. **(B)** Graph presents cell adhesion to inflamed aortic endothelium at days 1, 3 and 5 days. Representative images show monocyte adhesion to the endothelium by day 5 (*P < 0.05 compared to the time-matched sham-treated groups).

In the WT mice, the CLP-treated group showed elevated gene expression of proinflammatory cytokines compared to the sham-operated group (Figure 6A). TNF-α was upregulated 4.7-fold on day 3 (*P* = 0.014) and 2.7-fold on day 5 (*P* = 0.047), and the chemokine CCL2 was upregulated 6.1-fold on day 3 (*P* = 0.002) and 5.4-fold on day 5 (*P* < 0.001). Similarly, adhesion molecules were upregulated in the CLP group. ICAM-1 was upregulated 2.9-fold on day 1 (*P* = 0.01) and 2.3-fold on day 5 (*P* = 0.006). VCAM-1 gene expression was also upregulated, by 2.2-fold, though not until day 3 (*P* < 0.01) and day 5 (*P* < 0.001). Similar to the gene expression patterns, staining for ICAM-1 on endothelial luminal surfaces was prominent by day 5 compared to days 1 and 3 (Figure 6). VCAM-1 was also upregulated by day 5 and occasionally colocalized with ICAM-1 by day 5 (Figure 6A, inset). Using the functional *ex vivo* assay of endothelial activation, the CLP-treated mice had 1.7-fold higher monocyte adhesion by 3 days and 2.62-fold higher adhesion by 5 days (*P* = 0.03) (Figure 6B).

## Discussion

In this article, we present a combined approach used to interrogate whether acute sepsis accelerates chronic atherogenesis. We show that mice with preexisting atherosclerosis randomly allocated to a sepsis group had prolonged inflammation, increased atheroma burden, and greater macrophage infiltration into atheromatous plaques than sham-operated controls. Additionally, we show that sepsis induces acute inflammation in aortic tissues, increases endothelial monocyte adhesion and elevates markers of vascular inflammation [22]. This work suggests that, at least in a murine model, sepsis plays a causal role in accelerating both the total burden of atheroma and the infiltration of inflammatory cells into the plaques, possibly making the plaques more vulnerable to rupture. Importantly, the persistent effects did not appear to be due to ongoing infection. Thus, it seems probable that the accelerated atheroma is due to persistence of a local or systemic host inflammatory response.

Critical illness has long-lasting effects that continue beyond the time of hospitalization [23]. We and others have previously shown that cardiovascular disease is a leading cause of rehospitalization after community-acquired pneumonia (CAP), occurring in 19% over the course of 5 years [24]. Interestingly, only 23% of those who developed a cardiovascular event after severe sepsis had a prior history of clinically overt cardiovascular disease. In a separate cohort, CAP survivors had unresolved inflammation at hospital discharge despite being deemed ready for discharge, and higher IL-6 levels were associated with higher risk of cardiovascular disease–related deaths during the subsequent year [6]. As short-term mortality continues to decline with advances in critical care, there is an increasing focus on long-term morbidity and mortality. Our work suggests a causal role for sepsis in aggravating cardiovascular disease and thus raises the possibility of studying interventions in preclinical as well as clinical models and considering cardiovascular disease endpoints in sepsis trials.

Failure to gain weight or weight loss has been reported in previous murine sepsis studies, but only for shorter time horizons [25,26]. We were therefore intrigued by our finding of persistent failure to gain weight. Anorexia and weight loss are common in critical illness, prompting clinicians to engage in aggressive attempts to feed patients. Yet, weight loss has lowered atherosclerotic burden in both clinical and preclinical studies [27-29], whereas weight gain promotes atheroma [30]. Recently, King *et al*. demonstrated that atheroma burden grew in parallel with weight gain over a 24-week course in ApoE^−/−^ mice fed a high-fat diet [10]. Thus, both sepsis and weight gain promote atheroma. Consequently, had we been able to feed the mice enough to promote weight gain post-CLP treatment, the acceleration in atheroma may have been even worse. Furthermore, one wonders whether the anorexia following critical illness could have protective effects that are undermined by aggressive feeding practices.

There are several important considerations when interpreting our findings. First, to achieve our goal of testing for the effects of sepsis on cardiovascular disease, we had to forge a balanced combination of existing models. Combining the models required extensive pilot work to titrate the severity of the sepsis insult such that the animals were rendered appropriately sick, reflective of sepsis, and yet still likely to have high short-term survival with antibiotics and fluid resuscitation. Thus, for pragmatic reasons, we considered only one model of atherosclerosis and one model of sepsis [10,25].

Although we show a persistence of IL-6 and IL-10 elevations, their role in inflammation should be evaluated within the context of the stage of sepsis. IL-10 has been assigned a regulatory role, which, early in the course of sepsis, could improve the outcome, whereas it may be deleterious late in the course of sepsis, suggesting a dichotomous role for this cytokine [31,32].

The choice of atherosclerosis models is limited. Although a WT model would have been ideal, it is hard to induce atheroma in such animals. ApoE^−/−^ mice are prone to atheroma when fed a high-fat diet. However, obtaining, feeding and housing these mice for many months is expensive. Furthermore, knocking out ApoE could confound interpretation of the link between inflammation and atheroma, as ApoE modulates the type I inflammatory response and ApoE polymorphisms are associated with sepsis susceptibility and outcomes [33,34]. These reasons influenced our decision to use WT mice to assess acute inflammatory changes. To show additional parameters of inflammation, we will incorporate plasminogen activator inhibitor 1 and fibrinogen levels in future studies.

Ideally, we would have studied plaque rupture and thrombosis, but these events are rare in murine atheroma models. A brachiocephalic artery model or a cross between the ApoE^−/−^ mice and mice deficient in receptor class B, type I has promising results mimicking human disease and may be a better choice in future studies [35]. CLP is one of the most common models of sepsis, but may be indicative only of intra-abdominal sepsis. We do not know if alternative sepsis models, such as installation pneumonia models, would result in similar long-term effects. The optimal duration of follow-up is also unclear, although 5 months appeared adequately long to capture a variety of clinical and pathophysiologic changes. Additionally, extrapolating any murine data to humans requires caution, given poor cross-species correlation of the host response to infection and injury [36].

The purpose of this study was to determine whether sepsis plays a causal role in accelerating atheroma. However, the goal was not to determine the exact mechanism by which sepsis promotes atheroma. CLP induces a massive and complex host response, and the CLP insult itself, although a standardized injury, likely results in a variable pathogen load across animals. Thus, the number of plausible molecular and cellular pathways that could underlie the sepsis effects is huge, and their elucidation is beyond the scope of this article. Nevertheless, this model can serve as a translational tool when evaluating whether future therapies can modulate these late effects of sepsis.

## Conclusions

We developed a murine acute and chronic model combining abdominal sepsis and atherosclerosis mimicking patients surviving sepsis and succumbing to its downstream effects on atherosclerosis [37]. This model could be used to dissect the molecular mechanisms and test therapeutic strategies to improve long-term outcomes of sepsis.

## Key messages

- Acute sepsis preclinical models do not capture chronic changes; therefore, models mimicking long-term outcomes from sepsis are required to study human disease.
- We developed both acute and chronic sepsis survival models in mice to study vascular inflammation and progression of atherosclerosis.
- In a 5-day acute model, we show that the aorta becomes a site of inflammation with increased expression of ICAM-1, TNF-α, CCL2 and VCAM-1, as well as increased adhesion of monocytic cells to the endothelium.
- In the chronic model, which lasted for up to 9 months, we used atherosclerosis-prone ApoE^−/−^ mice. Over the course of the experimental period, sepsis-surviving mice had increased systemic inflammation and atheroma burden, whereas peritoneal bacterial cultures remained negative.
- In this study, we developed a chronic sepsis survival model with persistent inflammation and atherosclerosis in mice, making it possible to study clinically relevant questions in a preclinical model.

## Additional files

Additional file 1:
**Preliminary survival analysis with varying needle sizes.** There was only one dead mouse 7 days after CLP with a 25-gauge needle. Additional file 2:
**Collagen staining in the aortic root in association with sepsis on day 1 and at 3 and 5 months.** Bottom, representative images of aortic root collagen staining at 5 months.

## Abbreviations

ApoE^−/−^: Apolipoprotein E–deficient
CAP: Community-acquired pneumonia
CCL2: Chemokine (C-C motif) ligand 2
CLP: Cecal ligation and puncture
ICAM-1: Intercellular adhesion molecule 1
IL: Interleukin
MCP-1: Monocyte chemotactic protein 1
PBS: Phosphate-buffered saline
TNF-α: Tumor necrosis factor α
VCAM-1: Vascular cell adhesion molecule 1
WT: Wild type
