# Supplementary figures and images for: Effects of intra-abdominal sepsis on atherosclerosis in mice
Source: Crit Care. 2014 Sep 3;18(5):469. doi: 10.1186/s13054-014-0469-1 (PMC4172909; doi:10.1186/s13054-014-0469-1)

Additional File 1

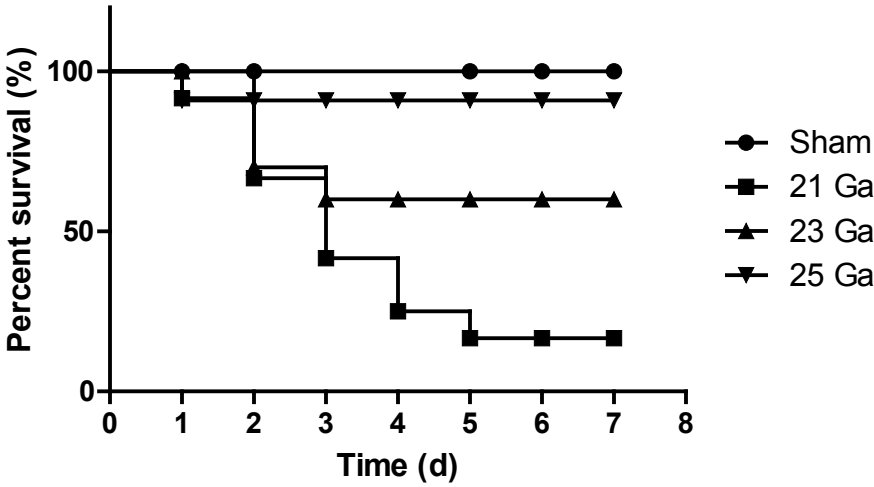

Supplement: Additional file 1: — Preliminary survival analysis with varying needle sizes. There was only one dead mouse 7 days after CLP with a 25-gauge needle. [file 13054_2014_469_MOESM1_ESM.pdf]

**Additional File 2**

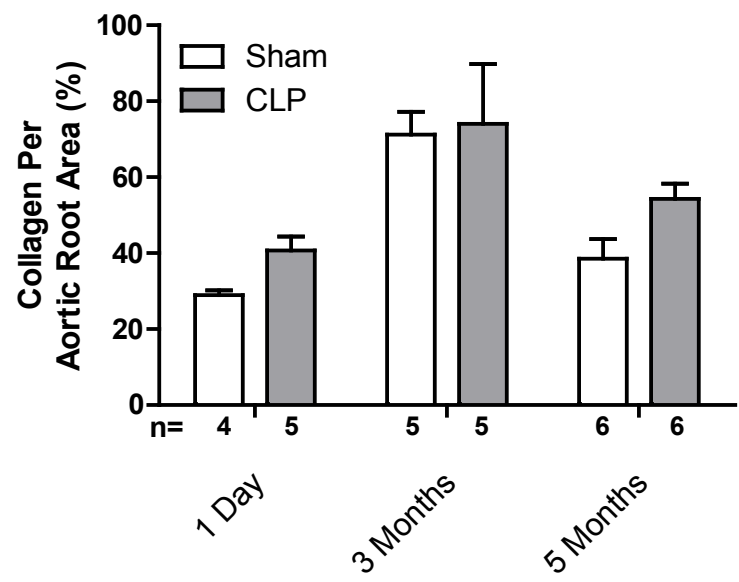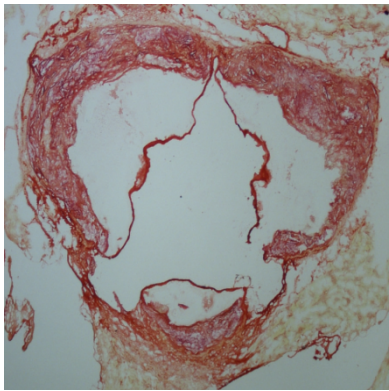

**Sham**

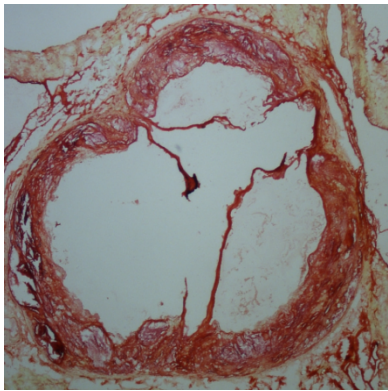

**CLP**

Supplement: Additional file 2: — Collagen staining in the aortic root in association with sepsis on day 1 and at 3 and 5 months. Bottom, representative images of aortic root collagen staining at 5 months. [file 13054_2014_469_MOESM2_ESM.pdf]
